# Remarks on Ambulant or Erratic Erysipelas

**Published:** 1845-03

**Authors:** 


*Remarks on Ambulant or Erratic Erysipelas.*—"The epithet applied to this form of Erysipelatous inflammation designates its characteristic feature. The cutaneous affection propagates itself by irregular impulsions. In one case it attacks a part which it speedily quits, without passing through its successive stages; and in another the disease exhibits all the phases of its complete evolution in the part that is primarily seized. Not obedient to any regular march, it leaps from one region to another, without implicating the intermediate parts; or it suddenly vanishes from the skin, to fasten itself upon some internal organ. The inflammatory action usually does not penetrate deep; it but skims the cutaneous surface. The danger, therefore, of this form of Erysipelas arises not so much from the extent or severity of the dermal lesions, as from the risk of metastasis of the morbid action to some important viscus, and from the cachectic state of the general health.

Erratic Erysipelas may terminate in various ways. In the majority of cases, it proves fatal. It very rarely terminates by simple resolution and by a direct return to health. A fortunate termination of the disease is usually accompanied or followed by some critical evacuation, as a hæmorrhage from the nose, a profuse perspiration, a copious flow of dark-colored urine, or by a severe diarrhœa.

Occasionally—but this termination has been but little attended to—the critical effort seems to consist in the development of numerous subcutaneous Abcesses, unpreceded by any inflammatory action in the parts so affected.

This lesion must not be confounded with the suppuration of the subcutaneous cellular tissue, that is not unfrequently the consequence of phlegmonous Erysipelas; in the ambulant form of the disease, the inflammation is strictly superficial, and the subjacent cellular substance is not involved in the morbid process.

In the two cases related by M. Tanquerel, erratic Erysipelas had continued for upwards of three weeks, and, being accompanied with Typhoid symptoms, threatened great danger; when, suddenly, a number of small indurated tubercles were perceptible under the skin of the neck and chest—the cutaneous inflammation having left these parts a few days before, and attacked the lower extremities. On the following day, many of these tumours presented a distinct sense of fluctuation. In one case, as many as forty-three of these boils or subcutaneous abscesses formed in the space of three weeks. The size of them varied from that of a small nut to that of a pigeon's egg.

They were developed without any previous heat, pain, or redness, in the parts affected; and even their existence was often not recognised, before it was found that purulent matter was already formed in them.

From the moment that the earliest abscess was formed, the Erysipelas ceased to make any further advance beyond the part where it happened then to be; and soon afterwards it ceased altogether.

We need scarcely say that the convalescence from such an attack must always be very tedious. There was no reason to suppose that, in either of the cases which occurred to Dr. T., there was any existing Phlebitis,—a lesion which is so often associated with, if it be not the immediate cause of, those *metastatic abscesses* which are generally referred to what has been called, of late years, a Purulent Infection of the System. It is more than probable that there is always present, in both sets of cases, a morbid alteration of the circulating fluids: although we are not yet prepared to say in what this alteration consists."—*Ibid, from Journal de Medecine.*

